# Postoperative outcomes in patients with COPD undergoing elective non-cardiac surgery: a propensity score-matched cohort study

**DOI:** 10.3389/fmed.2025.1641613

**Published:** 2025-11-07

**Authors:** Levan B. Berikashvili, Mariya M. Shemetova, Mikhail Ya Yadgarov, Kristina K. Kadantseva, Petr A. Polyakov, Alexey A. Yakovlev, Andrey G. Yavorovskiy, Valery V. Likhvantsev

**Affiliations:** 1 Department of Clinical Trials and Intelligent IT, Federal Research and Clinical Center of Intensive Care Medicine and Rehabilitology, Moscow, Russia; 2 Department of Anesthesiology, I.M. Sechenov First Moscow State Medical University, Moscow, Russia

**Keywords:** COPD, elective surgical procedure, non-cardiac anesthesia, hospital mortality, hospital stay, ICU mortality, ICU length of stay

## Abstract

**Background:**

Given the nature and pathophysiology of chronic obstructive pulmonary disease (COPD), it is reasonable to hypothesize that concomitant COPD may adversely affect clinical outcomes, leading to higher incidence of respiratory infections, prolonged mechanical ventilation, and prolonged hospital stay. However, robust evidence to support this assumption remains limited. The objective of this study was to evaluate the impact of chronic obstructive pulmonary disease (COPD) on postoperative outcomes in adult patients undergoing elective non-cardiac surgery.

**Methods:**

This retrospective cohort study analyzed data from the eICU Collaborative Research Database (eICU-CRD), including adult patients admitted to the ICU following elective non-cardiac surgery. Propensity score matching (PSM) was performed to adjust for confounding variables between COPD and non-COPD groups. Matching was based on age, sex, BMI, APACHE IV score, surgery type, and comorbidities. Post-matching outcomes included mortality, ICU/hospital length of stay, need for mechanical ventilation (MV), and postoperative lab parameters.

**Results:**

A total of 15,429 patients were included, with 1,720 (11.1%) having COPD. After PSM, 563 patients remained in each group. No significant differences were found in hospital (4.6% vs. 5.5%, *p* = 0.6) or ICU mortality (1.8% vs. 3.6%, *p* = 0.09). COPD patients had longer hospital stays (mean difference 1 day, *p* = 0.03) and a higher incidence of MV (35.9% vs. 27.7%, *p* = 0.003).

**Conclusion:**

Patients with chronic obstructive pulmonary disease (COPD) undergoing surgery demonstrate comparable ICU and hospital mortality rates to those without COPD. However, they tend to experience a longer hospital stay and require prolonged mechanical ventilation postoperatively.

## 1 Introduction

Globally, more than 300 million surgical procedures are performed annually, encompassing a wide spectrum of interventions, from minor outpatient procedures to highly complex surgeries (1). Of these, approximately two-thirds are classified as non-cardiac surgeries, a category that includes orthopedic, abdominal, thoracic (non-cardiac), vascular, and other types of interventions (1, 2). Despite ongoing advances in perioperative care, patient comorbidities remain a key determinant of surgical outcomes, often complicating both intraoperative and postoperative management.

Chronic Obstructive Pulmonary Disease (COPD) is a prevalent, progressive, and potentially life-threatening chronic respiratory condition characterized by airflow limitation that is not fully reversible (3, 4). It represents a significant public health challenge, particularly in the aging population, with a global prevalence estimated at over 10% in adults aged 40 and older (5). COPD is associated with systemic inflammation, impaired gas exchange, and decreased physiological reserve, all of which may negatively impact a patient's ability to respond to the physiological stress of surgery (6, 7). Therefore, it is reasonable to assume that patients with COPD undergoing elective non-cardiac surgery are at increased risk of perioperative complications, including prolonged mechanical ventilation, increased need for intensive care, higher incidence of respiratory infections, and extended hospital stays (8, 9).

However, upon closer examination of the existing literature, it becomes evident that high-quality evidence directly addressing the impact of COPD on postoperative outcomes in non-cardiac surgery is limited. While some observational studies suggest an increased risk of complications in this population, these findings are often confounded by other comorbidities, severity of illness, and variation in surgical and anesthetic techniques (10). Meta-analyses or randomized controlled trials focusing specifically on the surgical outcomes of patients with COPD, stratified by disease severity, remain scarce. The quality of evidence is further diluted by inconsistent definitions of COPD across studies, poor reporting of spirometric data, and lack of long-term follow-up.

Clinical guidelines do offer recommendations for the perioperative management of patients with COPD. For example, both the European Respiratory Society (ERS) and American Thoracic Society (ATS) suggest the use of non-invasive ventilation (NIV) as a strategy to prevent postoperative respiratory complications (11, 12). However, beyond this single strong recommendation, guidance remains largely empirical and is often extrapolated from studies in the intensive care setting or in patients with acute exacerbations rather than stable COPD undergoing elective surgery. This highlights a significant mismatch between the perceived risk attributed to COPD in surgical patients and the strength of evidence supporting specific perioperative interventions.

This discrepancy between the hypothesized increased risk and the lack of high-certainty data underscores the need for further research. The heterogeneity of existing studies and absence of standardized protocols for assessing and managing COPD patients in the surgical setting necessitate more robust methodologies. In this context, the use of advanced statistical tools, such as propensity score matching, can help minimize confounding and provide more accurate estimates of the impact of COPD on surgical outcomes.

The objective of the present study was to clarify the impact of COPD on postoperative outcomes in adult patients undergoing elective non-cardiac surgery using propensity score-matching analysis. By comparing well-matched cohorts of patients with and without COPD, we aimed to elucidate whether COPD independently contributes to postoperative morbidity and mortality and to identify potential targets for intervention.

## 2 Materials and methods

### 2.1 Data sources

The primary dataset employed in this study was the eICU Collaborative Research Database (eICU-CRD), developed by Philips Healthcare in collaboration with the Massachusetts Institute of Technology's Laboratory for Computational Physiology. The eICU-CRD contains comprehensive clinical data from 200,859 patient admissions across 335 intensive care units (ICUs) in 208 U.S. hospitals, collected during the period of 2014-2015 (13). All patient data were anonymized, negating the need for approval by a local ethics committee. One of the authors (M.Y.) successfully completed training modules on “Human Research: Data or Specimens Only Research”, and “Conflicts of Interest,” and was granted access to the eICU-CRD (eICU) database (certificate numbers: 56653575, 56653561, valid through June 21, 2026).

### 2.2 Selection criteria

This retrospective cohort study included all adult patients who underwent elective non-cardiac surgery and were subsequently admitted to the intensive care unit (ICU) from the operating room (OR), post-anesthesia care unit (PACU), or recovery room (RR). Exclusion criteria were: (1) ICU re-admissions; (2) patients aged 90 years or older, and (3) no data on hospital discharge status.

### 2.3 Data extraction

Data extraction was performed using SQLite version 3.45.2 (https://www.sqlite.org/) and DBeaver software. The extracted parameters included: (1) general patient information - sex, age, body mass index (BMI), acute physiology and chronic health evaluation (APACHE IV) score at admission, presence of COPD, and its severity (the “pastHistory” table, the “pastHistoryValueText” column); (2) surgical interventions; (3) comorbidities; (4) postoperative laboratory parameters (first ICU values); (5) postoperative complications, and (6) hospitalization outcomes.

### 2.4 Outcomes

The primary endpoint was in-hospital mortality. Secondary endpoints included ICU mortality, length of stay in the hospital and ICU, the requirement for mechanical ventilation (MV) and its duration in postoperative period, and the administration of vasoconstrictors. Data on the discharge location and postoperative laboratory parameters were also analyzed. Further, a subgroup analysis was performed by surgery type.

### 2.5 Statistical analysis

The distribution of the data was assessed using the Shapiro–Wilk test. Continuous variables were reported as medians (Me) with interquartile ranges (IQRs), while categorical variables were presented as frequencies and percentages (%). The chi-square test and Fisher's exact test were employed for comparing frequencies, and the Mann–Whitney U (Wilcoxon rank-sum) test was utilized for comparing continuous variables.

Given the confounding bias inherent to retrospective studies, a one-to-one propensity score matching analysis (PSM) was performed to adjust for unbalanced basic characteristics of COPD/no COPD groups. Logistic regression was used to develop propensity scores, 1:1 nearest neighbor method was used for matching (match tolerance [caliper] = 0.000006). We opted for matching to the sixth decimal point, as this value is less than 0.02 standard deviations (SDs) of the propensity score, which allowed us to achieve a satisfactory balance between the groups. The covariates included age, sex, BMI, APACHE IV score, surgery types, and various comorbidities (Table 1). We assessed the balance of covariates across groups by calculating standardized mean differences (SMDs) and visualized this using love plots, applying an SMD threshold of ≤ 0.1 as the success criterion. Additionally, we examined the balance of the propensity score distribution using balance plots.

**Table 1:** Initial parameters, surgery types and comorbidities before and after propensity score matching analysis.

**Parameters**	**Before matching**		***p*-value**	**After matching**		***p*-value**
	**No COPD**, ***N** =* **13,709**	**COPD**, ***N** =* **1,720**		**No COPD**, ***N** =* **563**	**COPD**, ***N** =* **563**	
Sex*^†^*(Male)	6,958, 50.8%	879, 51.1%	0.8^a^	273, 48.5%	265, 47.1%	0.6^a^
Age*^†^*, years	*N =* 13,709, 64.0 (IQR 52.0-73.0)	*N =* 1720, 69.0 (IQR 60.0-76.0)	**< 0.001** ^**b**^	*N =* 563, 68.0 (IQR 58.0-76.0)	*N =* 563, 66.0 (IQR 57.0-74.0)	0.093^b^
BMI*^†^*, kg/m^2^	*N =* 13,299, 27.9 (IQR 24.0-33.0)	*N =* 1677, 26.9 (IQR 22.7-32.2)	**< 0.001** ^**b**^	*N =* 563, 27.8 (IQR 24.0-32.7)	*N =* 563, 27.5 (IQR 23.1-34.1)	0.8^b^
APACHE IV*^†^*, score	*N =* 11,688, 44.0 (IQR 32.0-58.0)	*N =* 1560, 47.0 (IQR 37.0-62.0)	**< 0.001** ^**b**^	*N =* 563, 46.0 (IQR 34.0-58.0)	*N =* 563, 44.0 (IQR 35.0-60.0)	0.7^b^
Mild COPD	NA	631, 36.7%	NA	NA	209, 37.1%	0.9^a^ (before-after PSM)
Moderate COPD		853, 49.6%			273, 48.5%	
Severe COPD		236, 13.7%			81, 14.4%	
**Surgery types**						
Abdominal surgery*^†^*	3,877, 28.3%	548, 31.9%	**0.002** ^**a**^	195, 34.6%	193, 34.3%	0.9^a^
Thrombectomy under general anesthesia*^†^*	217, 1.6%	28, 1.6%	0.8^c^	11, 2%	7, 1.2%	0.5^c^
Head and neck surgery*^†^*	815, 5.9%	64, 3.7%	**< 0.001** ^**c**^	39, 6.9%	32, 5.7%	0.5^c^
Mastectomy*^†^*	58, 0.4%	4, 0.2%	0.2^c^	2, 0.4%	1, 0.2%	>0.9^c^
Neurological surgery*^†^*	2,845, 20.8%	192, 11.2%	**< 0.001** ^**a**^	95, 16.9%	91, 16.2%	0.7^a^
Orthopedic surgery*^†^*	356, 2.6%	52, 3%	0.3^c^	15, 2.7%	17, 3%	0.9^c^
Skin surgery*^†^*	467, 3.4%	42, 2.4%	**0.04** ^**c**^	10, 1.8%	16, 2.8%	0.3^c^
Thoracic surgery*^†^*	1,298, 9.5%	302, 17.6%	**< 0.001** ^**a**^	60, 10.7%	58, 10.3%	0.8^a^
Trauma surgery*^†^*	375, 2.7%	55, 3.2%	0.3^c^	7, 1.2%	12, 2.1%	0.4^c^
Urogenital surgery*^†^*	720, 5.3%	63, 3.7%	**0.005** ^**c**^	25, 4.4%	31, 5.5%	0.5^c^
Vascular surgery*^†^*	2,096, 15.3%	341, 19.8%	**< 0.001** ^**a**^	96, 17.1%	89, 15.8%	0.6^a^
Other*^†^*	408, 3%	28, 1.6%	**0.001** ^**c**^	8, 1.4%	16, 2.8%	0.2^c^
**Comorbidities**						
Anemia	64, 0.5%	13, 0.8%	0.14^c^	4, 0.7%	5, 0.9%	0.9^c^
Angina	143, 1%	55, 3.2%	**< 0.001** ^**c**^	7, 1.2%	12, 2.1%	0.4^c^
Arrhythmia*^†^*	883, 6.4%	224, 13%	**< 0.001** ^**c**^	32, 5.7%	47, 8.3%	0.1^c^
Arterial hypertension*^†^*	5821, 42.5%	1058, 61.5%	**< 0.001** ^**a**^	256, 45.5%	274, 48.7%	0.3^a^
Asthma	729, 5.3%	236, 13.7%	**< 0.001** ^**c**^	28, 5%	84, 14.9%	**< 0.001** ^**c**^
Chronic heart failure*^†^*	707, 5.2%	273, 15.9%	**< 0.001** ^**c**^	25, 4.4%	23, 4.1%	0.9^c^
Chronic kidney disease*^†^*	894, 6.5%	179, 10.4%	**< 0.001** ^**c**^	35, 6.2%	40, 7.1%	0.6^c^
Coronary artery bypass grafting	604, 4.4%	138, 8%	**< 0.001** ^**c**^	26, 4.6%	26, 4.6%	0.9^c^
Deep vein thrombosis*^†^*	349, 2.5%	68, 4%	**< 0.001** ^**c**^	14, 2.5%	18, 3.2%	0.6^c^
Heart valve disease	310, 2.3%	70, 4.1%	**< 0.001** ^**c**^	16, 2.8%	18, 3.2%	0.9^c^
Home oxygen	66, 0.5%	141, 8.2%	**< 0.001** ^**c**^	1, 0.2%	42, 7.5%	**< 0.001** ^**c**^
Hyperthyroidism	44, 0.3%	4, 0.2%	0.8^c^	4, 0.7%	3, 0.5%	>0.9^c^
Hypothyroidism	1,072, 7.8%	178, 10.3%	**< 0.001** ^**c**^	50, 8.9%	49, 8.7%	>0.9^c^
Insulin dependent diabetes*^†^*	1,033, 7.5%	178, 10.3%	**< 0.001** ^**c**^	53, 9.4%	53, 9.4%	>0.9^c^
Liver cirrhosis	199, 1.5%	23, 1.3%	0.8^c^	8, 1.4%	10, 1.8%	0.8^c^
Myocardial infarction*^†^*	693, 5.1%	166, 9.7%	**< 0.001** ^**c**^	32, 5.7%	30, 5.3%	0.9^c^
Neuromuscular disease*^†^*	94, 0.7%	6, 0.3%	0.11^c^	0, 0%	2, 0.4%	0.5^c^
Oncology	2,663, 19.4%	310, 18%	0.2^a^	111, 19.7%	104, 18.5%	0.6^a^
Peptic ulcer disease	251, 1.8%	66, 3.8%	**< 0.001** ^**c**^	9, 1.6%	19, 3.4%	0.08^c^
Peripheral vascular disease*^†^*	688, 5%	217, 12.6%	**< 0.001** ^**c**^	15, 2.7%	23, 4.1%	0.2^c^
Procedural coronary intervention	553, 4%	152, 8.8%	**< 0.001** ^**c**^	38, 6.7%	39, 6.9%	>0.9^c^
Pulmonary embolism	139, 1%	30, 1.7%	**0.007** ^**c**^	8, 1.4%	7, 1.2%	>0.9^c^
Respiratory failure	50, 0.4%	22, 1.3%	**< 0.001** ^**c**^	2, 0.4%	8, 1.4%	0.11^c^
Sarcoidosis	7, 0.1%	3, 0.2%	0.09^c^	0, 0%	1, 0.2%	>0.9^c^
Seizures	511, 3.7%	77, 4.5%	0.12^c^	16, 2.8%	25, 4.4%	0.2^c^
Stroke*^†^*	852, 6.2%	191, 11.1%	**< 0.001** ^**c**^	35, 6.2%	35, 6.2%	>0.9^c^

^a^Chi-square test; ^b^Mann–Whitney U test; ^c^Fisher's exact test.

^†^Covariates included in propensity score matching.

APACHE IV, Acute Physiology and Chronic Health Evaluation IV; BMI, body mass index; COPD, chronic obstructive pulmonary disease; NA, not applicable; PSM, propensity score matching.

Bold font indicates statistical significance.

A two-sided significance level was set at 0.05. All statistical analyses were performed using IBM SPSS Statistics v. 29.0 with fuzzy STATS_PSM extension for PSM analysis. To generate the love plot and balance plot, we used Python v. 3.12.5 with the modules including matplotlib, pandas, numpy, and seaborn.

## 3 Results

### 3.1 Patient characteristics

This study was conducted and reported in accordance with strengthening the reporting of observational studies in epidemiology (STROBE) guidelines (Supplementary Appendix 1). A total of 2,024 patients were sequentially excluded based on the specified criteria, resulting in a final cohort of 15,429 patients (50.8% males, median age 64 years [IQR 52-73]), of whom 1,720 (11.1%) had COPD.

**Figure 1:**
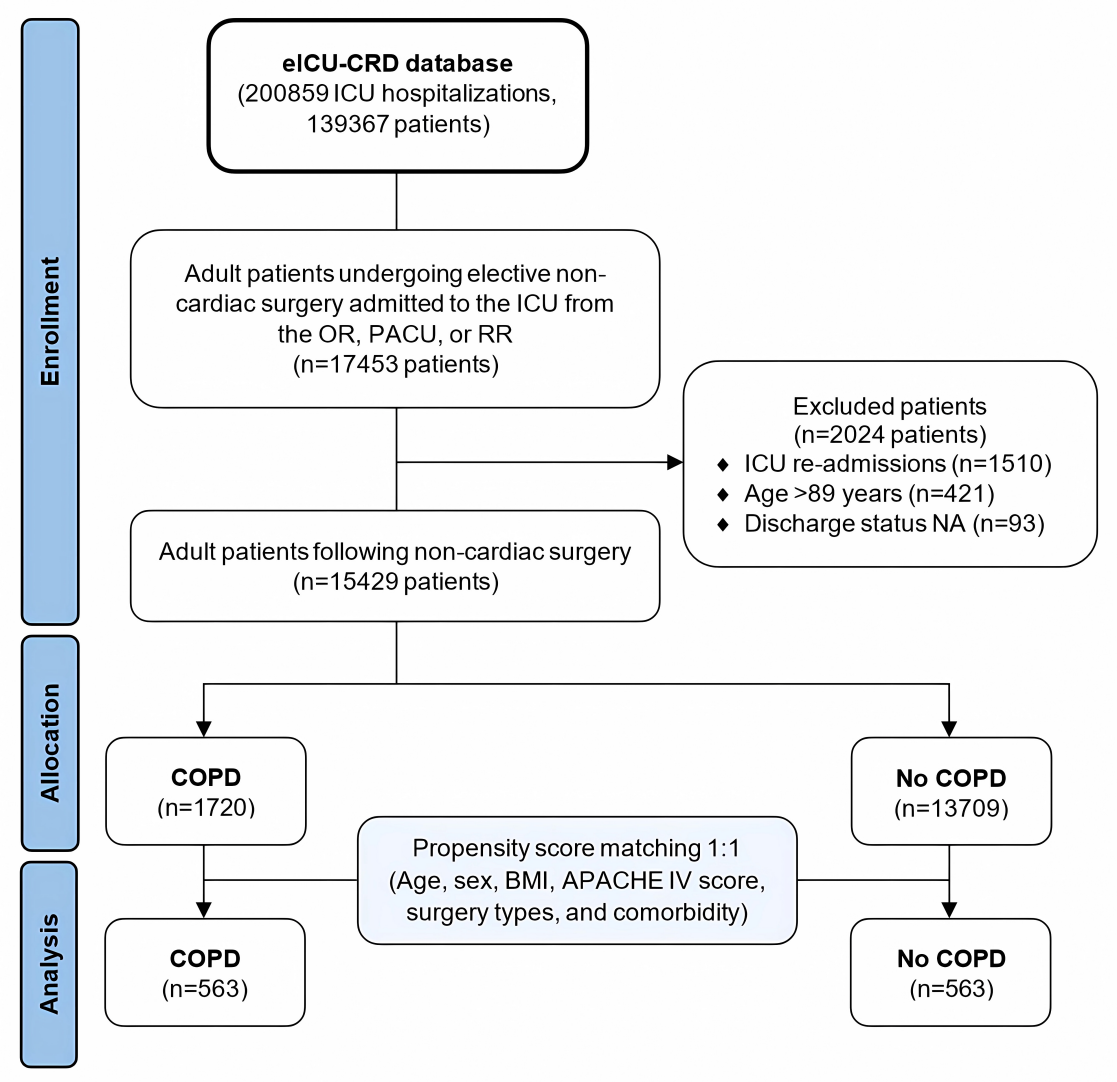
Flowchart of patient selection in the study.

A flowchart illustrating the patient selection process is presented in Figure 1.

Before propensity score matching, significant differences were observed between the COPD and non-COPD groups in age (median 69 vs. 64 years, *p* < 0.001), BMI (median 26.9 vs. 27.9 kg/m^2^, *p* < 0.001), APACHE IV score (median 47 vs. 44, *p* < 0.001), surgery types, and most comorbidities (Table 1).

**Figure 2:**
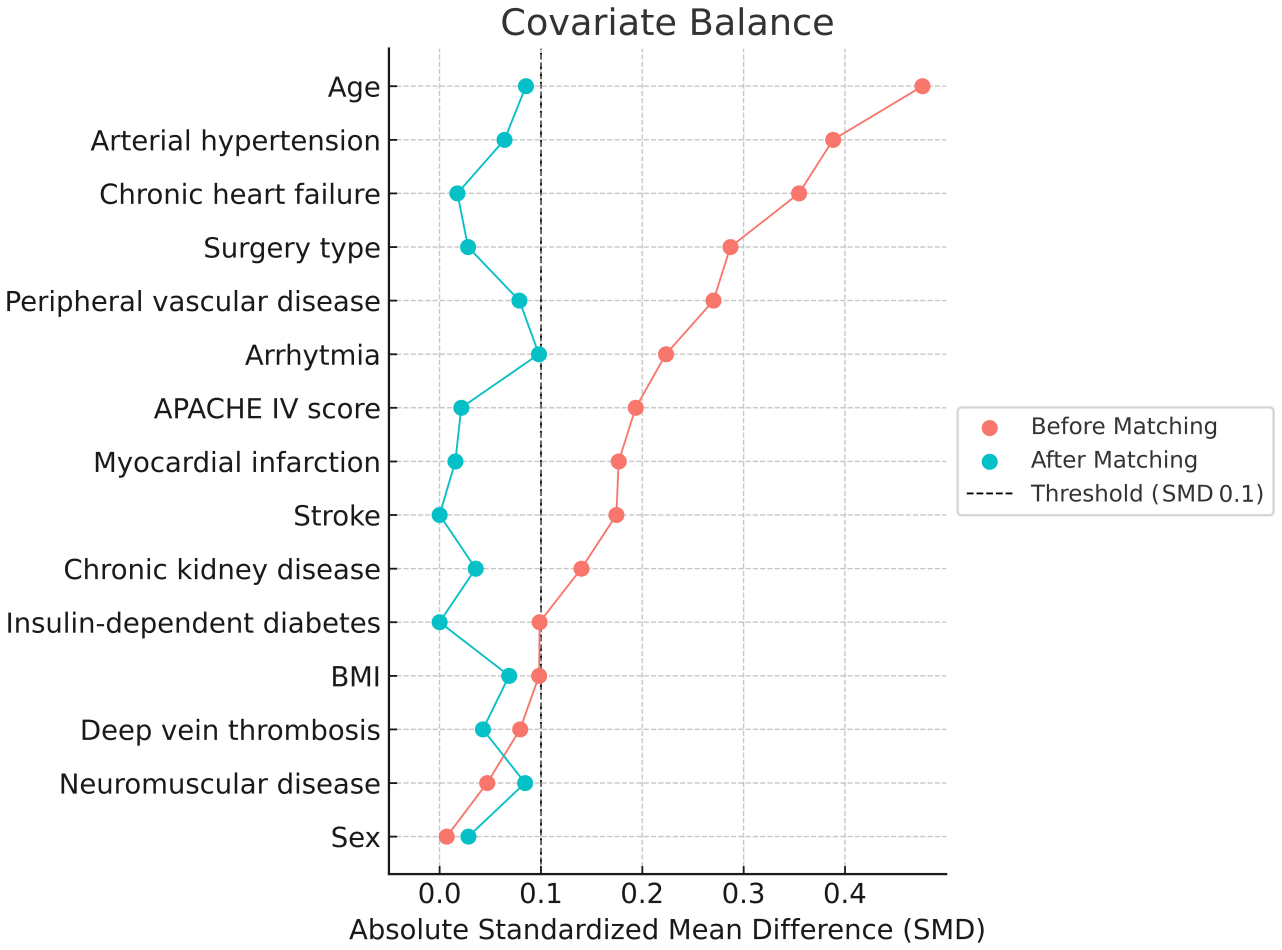
Love plot before (red color) and after (green color) propensity score matching (PSM) among selected covariates. The distribution was stable after matching.

**Figure 3:**
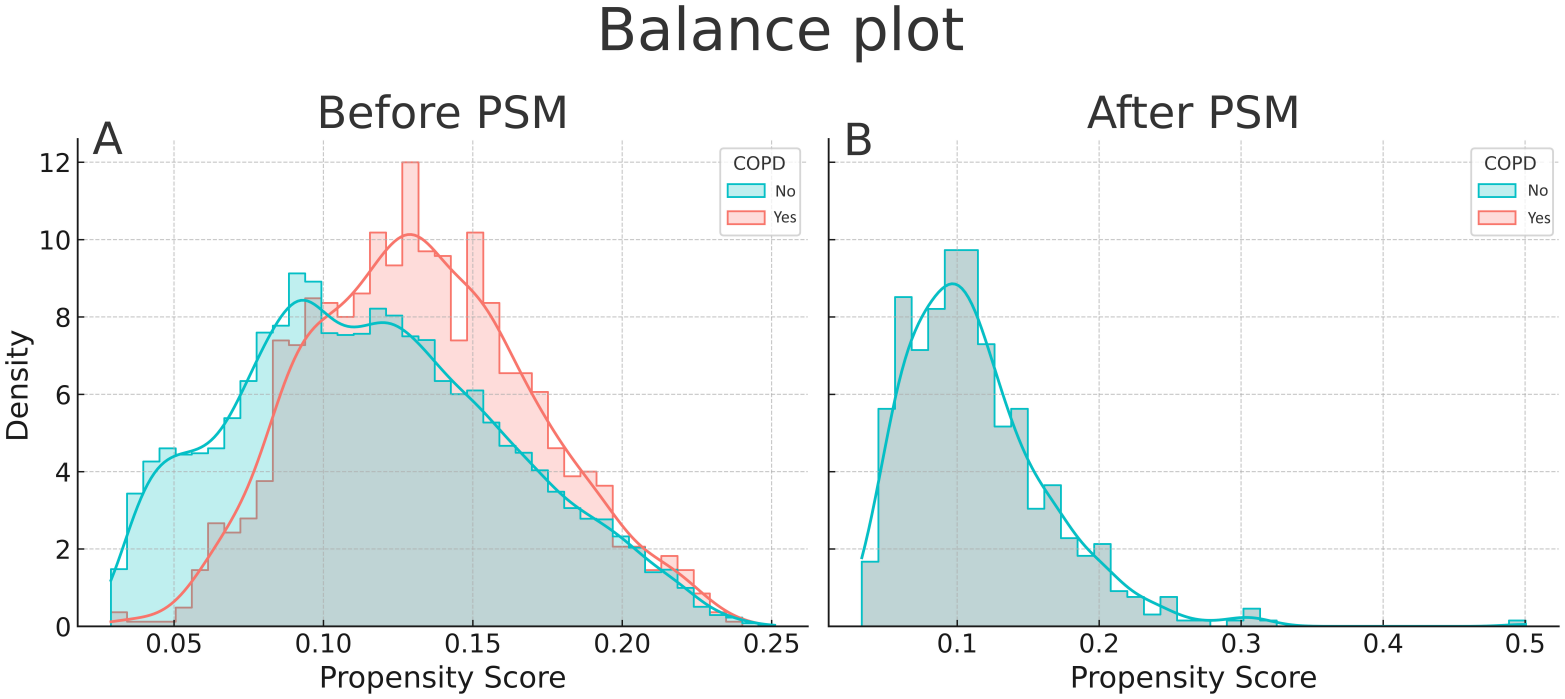
Balance plot before **(A)** and after **(B)** propensity matching (PSM).

After PSM, 563 patients remained in each group, achieving an adequate balance across all covariates (Figures 2, 3). The median age of the matched cohort was 67 years (IQR 47–75), with an age range of 19 to 89 years. 48.5% of patients had moderate COPD, 37.1% had mild COPD, and 14.4% had severe COPD. The most common surgical procedures were abdominal (34%), thoracic (10.5%), and vascular surgeries (16%). More than 45% of the patients had arterial hypertension. Among those with COPD, 7.5% required home oxygen therapy, and 15% had a history of asthma. Additionally, a significant proportion of the overall cohort (19%) had oncological conditions (Table 1).

### 3.2 Propensity-matched analysis

The fully adjusted PSM analysis for studied outcomes is shown in Table 2.

**Table 2:** Postoperative laboratory parameters, complications, hospitalization outcomes and discharge location after propensity score matching analysis.

**Parameters**	**No COPD, *N =* 563**	**COPD, *N =* 563**	***p-*value**
**Hospitalizations outcomes**			
Hospital mortality	31, 5.5%	26, 4.6%	0.6^b^
ICU mortality	20, 3.6%	10, 1.8%	0.09^b^
Duration of hospitalization, days	*N =* 563, 5.6 (IQR 2.1-8.8)	*N =* 563, 5.7 (IQR 3-10.1)	**0.03** ^**a**^
Length of ICU stay, days	*N =* 563, 1.4 (IQR 1-2.8)	*N =* 563, 1.6 (IQR 0.9-2.9)	0.5^a^
Need for MV	156, 27.7%	202, 35.9%	**0.003** ^**c**^
Duration of MV, days	*N =* 156, 2 (IQR 1-3)	*N =* 202, 2 (IQR 1-3)	0.7^a^
Use of vasoactive drugs	61, 10.8%	58, 10.3%	0.8^c^
**Laboratory parameters**			
Glucose, mg/dL	*N =* 508, 142 (IQR 118.5-175)	*N =* 521, 140 (IQR 114-169)	0.2^a^
Hgb, g/dL	*N =* 520, 10.9 (IQR 9.7-12)	*N =* 526, 11.3 (IQR 9.8-12.7)	**0.011** ^**a**^
Hct, %	*N =* 519, 32.9 (IQR 29.4-36.3)	*N =* 528, 34.2 (IQR 30.2-38.5)	**< 0.001** ^**a**^
Creatinine, mg/dL	*N =* 515, 0.9 (IQR 0.7-1.2)	*N =* 526, 0.8 (IQR 0.7-1.2)	0.2^a^
BUN, mg/dL	*N =* 510, 14 (IQR 10-21)	*N =* 524, 15 (IQR 10-21)	0.5^a^
Platelets, K/mcL	*N =* 512, 203 (IQR 156-255)	*N =* 520, 211 (IQR 160.5-269.5)	0.12^a^
WBC, K/mcL	*N =* 515, 11 (IQR 8.3-14.6)	*N =* 521, 12.6 (IQR 9-16.2)	**< 0.001** ^**a**^
RBC, M/mcL	*N =* 515, 3.6 (IQR 3.3-4.1)	*N =* 519, 3.8 (IQR 3.3-4.3)	**0.001** ^**a**^
Albumin, g/dL	*N =* 299, 2.7 (IQR 2.3-3.2)	*N =* 305, 2.8 (IQR 2.3-3.2)	0.4^a^
AST, Units/L	*N =* 277, 29 (IQR 19-63)	*N =* 290, 26 (IQR 17-44)	**0.006** ^**a**^
ALT, Units/L	*N =* 274, 26.5 (IQR 14-50)	*N =* 285, 21 (IQR 14-34)	**0.007** ^**a**^
Total protein, g/dL	*N =* 274, 5.5 (IQR 4.9-6.1)	*N =* 287, 5.6 (IQR 4.9-6.3)	0.18^a^
Total bilirubin, mg/dL	*N =* 268, 0.7 (IQR 0.4-1.1)	*N =* 283, 0.6 (IQR 0.4-0.9)	0.09^a^
Lactate, mmol/L	*N =* 113, 1.7 (IQR 1.2-3.5)	*N =* 115, 1.7 (IQR 1.1-2.8)	0.2^a^
Troponin I, ng/mL	*N =* 54, 0 (IQR 0-0.1)	*N =* 49, 0 (IQR 0-0.1)	0.8^a^
Troponin T, ng/mL	*N =* 9, 0.1 (IQR 0.1-0.1)	*N =* 10, 0 (IQR 0-0.2)	0.5^a^
pH	*N =* 183, 7.4 (IQR 7.3-7.4)	*N =* 220, 7.4 (IQR 7.3-7.4)	0.6^a^
CPK, Units/L	*N* = 37, 196 (IQR 74-684)	*N* = 57, 102 (IQR 61-313)	0.12^a^
Fibrinogen, mg/dL	*N =* 37, 283 (IQR 212-445)	*N =* 55, 315 (IQR 217-474)	0.6^a^
**Complications**			
Heart block and ventricular arrhythmias	2, 0.4%	0, 0%	0.5^b^
Heart failure	7, 1.2%	3, 0.5%	0.3^b^
Stroke	4, 0.7%	4, 0.7%	>0.9^b^
Acute kidney injury	23, 4.1%	26, 4.6%	0.8^b^
ARDS	2, 0.4%	4, 0.7%	0.7^b^
Respiratory failure	28, 5%	29, 5.2%	>0.9^b^
Pleural effusion	4, 0.7%	2, 0.4%	0.7^b^
Arrhythmia	21, 3.7%	24, 4.3%	0.8^b^
Sepsis	8, 1.4%	6, 1.1%	0.8^b^
Septic shock	6, 1.1%	11, 2%	0.3^b^
Thrombosis	3, 0.5%	3, 0.5%	>0.9^b^
Multiorgan failure	1, 0.2%	1, 0.2%	>0.9^b^
**Discharge location**			
Home	396, 70.3%	369, 65.5%	0.09^c^
Other (rehabilitation, other hospital)	167, 29.7%	194, 34.5%	

Quantitative data are presented in N, Me (IQR) format.

^a^Mann–Whitney U test; ^b^Fisher's exact test; ^c^chi-square test.

ALT, alanine aminotransferase; APACHE IV, Acute Physiology and Chronic Health Evaluation IV; AST, aspartate aminotransferase; BMI, body mass index; BUN, blood urea nitrogen; CI, confidence interval; COPD, chronic obstructive pulmonary disease; CPK, creatine phosphokinase; Hct, hematocrit; Hgb, hemoglobin; ICU, intensive care unit; IQR, interquartile range; ICU, intensive care unit; MV, mechanical ventilation; ND, no data; RBC, red blood cell count; WBC, white blood cell count.

Bold font indicates statistical significance.

No significant differences were observed in hospital mortality (4.6% in the COPD group vs. 5.5% in the non-COPD group, *p* = 0.6) or ICU mortality (1.8% in the COPD group vs. 3.6% in the non-COPD group, *p* = 0.09). The median ICU stay duration was similar between groups, with 1.6 days (IQR 0.9-2.9) for the COPD group and 1.4 days (IQR 1.0-2.8) for the non-COPD group (*p* = 0.5). However, patients with COPD had significantly longer hospital stays, with a median of 5.7 days (IQR 3.0-10.1) compared to median of 5.6 days (IQR 2.1-8.8) for those without COPD (*p* = 0.03) (Table 2).

Postoperative mechanical ventilation was required in 358 patients (32%), with a higher incidence in the COPD group (35.9%) compared to the non-COPD group (27.7%) (*p* = 0.003). The median duration of MV was 2 days (IQR 1-3), with no significant difference between the groups (*p* = 0.7). Vasoactive drugs after surgery were administered to 130 patients (12%), with comparable usage between the two groups (*p* = 0.8). About one-third of patients in both groups were transferred to another hospital for further treatment or rehabilitation post-discharge.

Patients with COPD demonstrated significantly higher postoperative hemoglobin (Hgb) levels (*p* = 0.011), hematocrit (Hct) levels (p < 0.001), as well as elevated WBC (*p* < 0.001) and RBC counts (*p* = 0.001). In contrast, they exhibited lower levels of AST (*p* = 0.006) and ALT (*p* = 0.007). The incidence of postoperative complications was comparable between the two groups (Table 2).

### 3.3 Subgroup analysis

In an additional subgroup analysis by type of surgery, patients with COPD who underwent thoracic or vascular surgery had significantly longer hospital stays (median 8.0 days vs. median 5.9 days, *p* = 0.017 and median 1.9 days vs. 1.4 days, *p* = 0.038, respectively), as well as extended ICU stays in the thoracic surgery group (median 2.0 days vs. 1.2 days, *p* = 0.034) (Table 3). The need for MV was higher in COPD patients in the abdominal surgery group (54.9% vs. 44.6%, *p* = 0.042).

**Table 3:** Subgroup analysis – COPD and hospitalization outcomes by main surgery types.

**Parameters**	**No COPD, *N =* 563**	**COPD, *N =* 563**	***p*-value**
**Abdominal surgery**, ***N** =* **388 (No COPD: 195, COPD: 193)**			
Hospital mortality	19, 9.7%	18, 9.3%	>0.9^a^
ICU mortality	15, 7.7%	7, 3.6%	0.12^a^
Duration of hospitalization, days	7.8 (IQR 5.8-11.8)	8.8 (IQR 5.3-14.2)	0.3^b^
Length of ICU stay, days	2 (IQR 1.1-3.9)	1.9 (IQR 1.1-3.8)	0.8^b^
Need for MV	87, 44.6%	106, 54.9%	**0.042** ^**c**^
Duration of MV, days	*N =* 87, 2 (IQR 1-3)	*N =* 106, 2 (IQR 1-3)	0.5^b^
Use of vasoactive drugs	34, 17.4%	31, 16.1%	0.7^c^
**Neurological surgery**, ***N** =* **186 (No COPD: 95, COPD: 91)**			
Hospital mortality	2, 2.1%	3, 3.3%	0.7^a^
ICU mortality	1, 1.1%	1, 1.1%	>0.9^a^
Duration of hospitalization, days	3.7 (IQR 2-5.8)	3.8 (IQR 2.1-6.7)	0.3^b^
Length of ICU stay, days	1.1 (IQR 0.9-2.8)	1.1 (IQR 0.9-2.1)	0.9^b^
Need for MV	12, 12.6%	11, 12.1%	0.9^c^
Duration of MV, days	*N =* 12, 2 (IQR 1-8.5)	*N =* 11, 2 (IQR 1-3)	0.6^b^
Use of vasoactive drugs	4, 4.2%	4, 4.4%	>0.9^a^
**Thoracic surgery**, ***N** =* **118 (No COPD: 60, COPD: 58)**			
Hospital mortality	1, 1.7%	2, 3.4%	0.6^a^
ICU mortality	0, 0%	0, 0%	NA
Duration of hospitalization, days	5.9 (IQR 4.2-8)	8 (IQR 4.9-13.6)	**0.017** ^**b**^
Length of ICU stay, days	1.2 (IQR 0.9-2.1)	2 (IQR 0.9-4.2)	**0.034** ^**b**^
Need for MV	14, 23.3%	17, 29.3%	0.5^c^
Duration of MV, days	*N =* 14, 2 (IQR 1-2)	*N =* 17, 2 (IQR 2-6)	0.12
Use of vasoactive drugs	6, 10.0%	4, 6.9%	0.7^a^
**Vascular surgery**, ***N** =* **185 (No COPD: 96, COPD: 89)**			
Hospital mortality	1, 1.0%	0, 0%	>0.9^a^
ICU mortality	0, 0%	0, 0%	NA
Duration of hospitalization, days	1.4 (IQR 1-2.9)	1.9 (IQR 1-4.9)	**0.038** ^**b**^
Length of ICU stay, days	1 (IQR 0.9-1.2)	1 (IQR 0.9-1.8)	0.3^b^
Need for MV	3, 3.1%	5, 5.6%	0.4^c^
Duration of MV, days	*N =* 3, 2 (IQR 2-2)	*N =* 5, 2 (IQR 1-2)	0.4^b^
Use of vasoactive drugs	6, 6.3%	5, 5.6%	>0.9^a^
**Other surgery**, ***N** =* **249 (No COPD: 117, COPD: 132)**			
Hospital mortality	8, 6.8%	3, 2.3%	0.12^a^
ICU mortality	4, 3.4%	2, 1.5%	0.4^a^
Duration of hospitalization, days	5.7 (IQR 2.9-9.8)	5 (IQR 3.6-9.7)	>0.9^b^
Length of ICU stay, days	1.8 (IQR 1-3.1)	1.5 (IQR 1-2.6)	0.2^b^
Need for MV	40, 34.2%	63, 47.7%	**0.03** ^**c**^
Duration of MV, days	*N =* 40, 2 (IQR 1-3)	*N =* 63, 2 (IQR 1-3)	0.8^b^
Use of vasoactive drugs	11, 9.4%	14, 10.6%	0.8^c^

Quantitative data are presented in N, Me (IQR) format.

^a^Fisher's exact test; ^b^Mann–Whitney U test; ^c^chi-square test.

ICU, intensive care unit; MV, mechanical ventilation; NA, not applicable; IQR, interquartile range; COPD, chronic obstructive pulmonary disease.

Bold font indicates statistical significance.

## 4 Discussion

### 4.1 Key findings

This retrospective cohort study analyzed the impact of COPD on postoperative outcomes in adult patients following elective non-cardiac surgery, using data from the eICU Collaborative Research Database. After PSM to balance baseline characteristics, 563 patients with COPD were compared with 563 patients without COPD. No significant differences in hospital or ICU mortality were observed between the groups; however, patients with COPD experienced significantly longer hospital stays. Additionally, the incidence of MV was notably higher in the COPD group, particularly among those undergoing abdominal surgery.

Patients with COPD exhibited higher postoperative hemoglobin, hematocrit, WBC, and RBC counts, but lower AST and ALT levels. Subgroup analysis revealed that patients with COPD who underwent thoracic or vascular surgeries had extended hospital stays, and ICU stays (for thoracic surgery).

### 4.2 Relationship with previous studies

Our findings align with and expand upon existing literature examining the perioperative outcomes of patients with COPD. Prior studies have consistently reported increased postoperative complications, prolonged hospital stays, and a higher likelihood of requiring mechanical ventilation in surgical patients with COPD, particularly following major procedures such as thoracic and abdominal surgeries (8, 14). However, our results suggest that while COPD patients had increased ventilatory requirements and longer hospital stays, there was no significant difference in ICU or hospital mortality compared to non-COPD patients.

The observed increased postoperative mechanical ventilation rates in COPD patients are consistent with previous findings, which highlight impaired pulmonary mechanics, diminished respiratory reserve, and an increased susceptibility to postoperative respiratory failure in this population (15). Nevertheless, despite this increased ventilatory burden, the duration of mechanical ventilation in our study was comparable between COPD and non-COPD patients, which may reflect improved perioperative management strategies, including lung-protective ventilation, goal-directed fluid therapy, and the optimization of bronchodilator regimens.

Several studies have also reported that COPD is associated with prolonged hospital length of stay following major surgery (16, 17). Our study corroborates these findings, demonstrating that COPD patients who underwent thoracic and vascular surgeries had a significantly longer hospital stay. This may be attributed to persistent postoperative pulmonary dysfunction, higher rates of exacerbations, and increased postoperative monitoring requirements.

Interestingly, our study did not find a significant increase in overall postoperative complications among COPD patients, which contrasts with some earlier reports (18, 19). This discrepancy may be due to differences in COPD severity or other selection criteria for surgical candidates in contemporary practice. Additionally, the absence of a significant difference in mortality between groups is noteworthy, as previous studies have suggested that COPD is an independent predictor of mortality in surgical populations (20). The observed variations in the impact of COPD on postoperative outcomes can be primarily attributed to the use of a propensity score matching approach. Matching COPD and non-COPD cohorts based on a set of relevant covariates was essential to isolate the specific effect of COPD on perioperative mortality. However, this finding does not contradict the established evidence of higher long-term mortality among COPD patients, particularly in cases with an increased frequency of exacerbations (21).

Overall, our findings emphasize that current perioperative management protocols are effective in mitigating the negative impact of COPD on surgical outcomes. However, further research is needed to elucidate the relationship between COPD severity and perioperative outcomes.

### 4.3 Strengths and limitations

The key strength of this study is the use of a large, comprehensive dataset from the eICU-CRD, which includes a diverse, multicenter cohort of patients across various ICUs in the United States. This multicentric approach enhances the external validity and generalizability of our findings. Additionally, the application of PSM effectively minimized confounding, allowing for a more balanced comparison between patients with and without COPD. The inclusion of subgroup analyses further strengthens the study, providing a detailed examination of postoperative outcomes by surgical type.

However, several limitations should be acknowledged. Due to the relatively small caliper used in the PSM analysis, some data were inevitably lost, potentially reducing the statistical power of the study. The retrospective nature of this study limits causal inference and introduces the possibility of selection bias and misclassification. While PSM was employed to control for multiple confounders, residual confounding cannot be entirely ruled out. Furthermore, it is important to note that the eICU-CRD database contains data from the 2014-2015 period, which may represent a study limitation. However, there have been no substantial changes in perioperative management strategies for COPD patients since that time. Therefore, the findings of this study remain clinically relevant and current.

### 4.4 Significance of study findings

Our study provides important insights into the perioperative outcomes of critically ill surgical patients with COPD, reinforcing key considerations for anesthesiologists and intensivists managing this high-risk population. While COPD is often associated with increased perioperative morbidity, our findings suggest that modern perioperative strategies may be mitigating its impact on mortality and major complications.

The observed increase in postoperative mechanical ventilation rates among COPD patients underscores the need for vigilant perioperative respiratory management. Optimized ventilatory strategies—including lung-protective ventilation, individualized positive end-expiratory pressure (PEEP) settings, and early extubation protocols—may be critical in reducing the risk of prolonged ventilatory dependence in these patients.

The longer hospital stays observed in COPD patients, particularly in those undergoing thoracic and vascular surgeries, highlight the potential for increased resource utilization and prolonged recovery in this population. This finding emphasizes the importance of preoperative risk stratification and multidisciplinary care approaches, including pulmonary rehabilitation, smoking cessation programs, and tailored anesthetic techniques, to optimize perioperative outcomes.

Our study also contributes to the growing body of evidence that COPD does not necessarily confer an increased risk of perioperative mortality when contemporary perioperative management strategies are employed. This challenges traditional perceptions that COPD is invariably associated with poor surgical outcomes and suggests that anesthetic and critical care advancements are improving the prognosis for these patients.

Ultimately, our findings highlight the evolving role of anesthesiologists in perioperative optimization, emphasizing the importance of individualized respiratory management, multimodal analgesia, and enhanced recovery protocols. Further research should explore targeted interventions—such as prehabilitation programs and advanced ventilatory strategies—that may further reduce the impact of COPD on postoperative outcomes.

### 4.5 Future studies and prospects

Building upon our findings, future research should focus on identifying specific perioperative interventions that can further improve outcomes for patients with COPD undergoing surgery. Investigating the efficacy of preoperative pulmonary rehabilitation programs tailored to COPD patients could be beneficial, as such interventions have shown promise in enhancing respiratory function and reducing length of hospital stay (22). However, feasibility studies have highlighted challenges in patient identification and recruitment, suggesting a need for earlier diagnosis and integration of COPD management in the surgical pathway (23).

Additionally, exploring the role of perioperative bronchodilator therapy in preserving lung function post-surgery is warranted. Recent studies indicate that such therapies may be effective in maintaining pulmonary function in COPD patients undergoing procedures like non-small cell lung cancer surgery (24). Further research could determine the optimal patient-specific bronchodilator regimens and their impact on a broader range of surgical procedures.

Moreover, the development of predictive models to stratify risk in COPD patients undergoing various types of surgery could aid in personalized perioperative care. By identifying patients at higher risk for complications, targeted interventions can be implemented to mitigate these risks. Prospective studies are needed to validate such models and assess their effectiveness in clinical practice.

## 5 Conclusion

Our study reveals that while patients with chronic obstructive pulmonary disease (COPD) undergoing surgery experience higher rates of postoperative mechanical ventilation and extended hospital stays, their ICU and hospital mortality rates are comparable to those without COPD. This suggests that current perioperative management strategies may effectively mitigate some risks traditionally associated with COPD in surgical settings. However, the increased resource utilization observed underscores the need for continued optimization of perioperative care in this population.

## Data Availability

The original contributions presented in the study are included in the article/Supplementary material, further inquiries can be directed to the corresponding author.
